# Long-Term Antithrombotic Therapy After Percutaneous Coronary Intervention: Secondary Prevention Beyond One Year

**DOI:** 10.31083/RCM51947

**Published:** 2026-07-24

**Authors:** Mohamed Abdelgader, Roann Khalid, Kartik Yadav, Sama Ehab Salah Ahmed, Murugapathy Veerasamy, Arka Das, Heerajnarain Bulluck

**Affiliations:** ^1^Department of Cardiology, Leeds Teaching Hospitals NHS Trust, LS1 3EX Leeds, UK; ^2^General Medicine, King’s College Hospital NHS Foundation Trust, SE5 9RS London, UK; ^3^Leeds Institute of Cardiovascular and Metabolic Medicine, University of Leeds, LS2 9JT Leeds, UK

**Keywords:** aspirin, clopidogrel, percutaneous coronary intervention, monotherapy, secondary prevention, rivaroxaban, pharmacogenetics, colchicine, icosapent ethyl, thrombosis

## Abstract

The early period following percutaneous coronary intervention (PCI), particularly the first days to weeks after stent implantation, is characterized by heightened device-related thrombotic risk, and dual antiplatelet therapy (DAPT) during this phase is well established. With contemporary drug-eluting stents (DES) and abbreviated DAPT strategies, late stent-related events have become less frequent, and long-term risk increasingly reflects underlying systemic atherosclerotic disease. Beyond 12 months, the risk profile shifts further: late events increasingly reflect progressive atherosclerosis and systemic vascular disease rather than stent-related thrombosis. This review synthesizes contemporary evidence on long-term antiplatelet and antithrombotic therapy after PCI, with a focus on the period beyond one year. We evaluate the data supporting for lifelong aspirin as the traditional default strategy, as well as the emerging evidence that P2Y_12_ inhibitor monotherapy may be superior to aspirin in selected post-PCI populations (HOST-EXAM, HOST-EXAM Extended, PANTHER, SMART-CHOICE 3). This review also examines the role of prolonged DAPT in selected high-risk patients (DAPT Trial, PEGASUS–Thrombolysis in Myocardial Infarction (TIMI) 54) and the paradigm of dual-pathway inhibition with low-dose rivaroxaban plus aspirin (COMPASS, VOYAGER-peripheral artery disease (PAD)). In addition, the review considers protease-activated receptor (PAR) antagonism (TRA 2°P–TIMI 50) and precision-based strategies, including *CYP2C19* genotype-guided therapy. Special populations, such as patients with diabetes, chronic kidney disease (CKD), polyvascular disease, and those requiring concomitant oral anticoagulation, present distinct long-term management challenges. Thus, novel targets, including factor XIa inhibitors and PAR-4 antagonists, may further refine the antithrombotic approach. Overall, the optimal long-term strategy after PCI is increasingly defined not by fixed timelines but by the dynamic balance between ischemic and bleeding risk.

## 1. Introduction

**Fig. 1.**
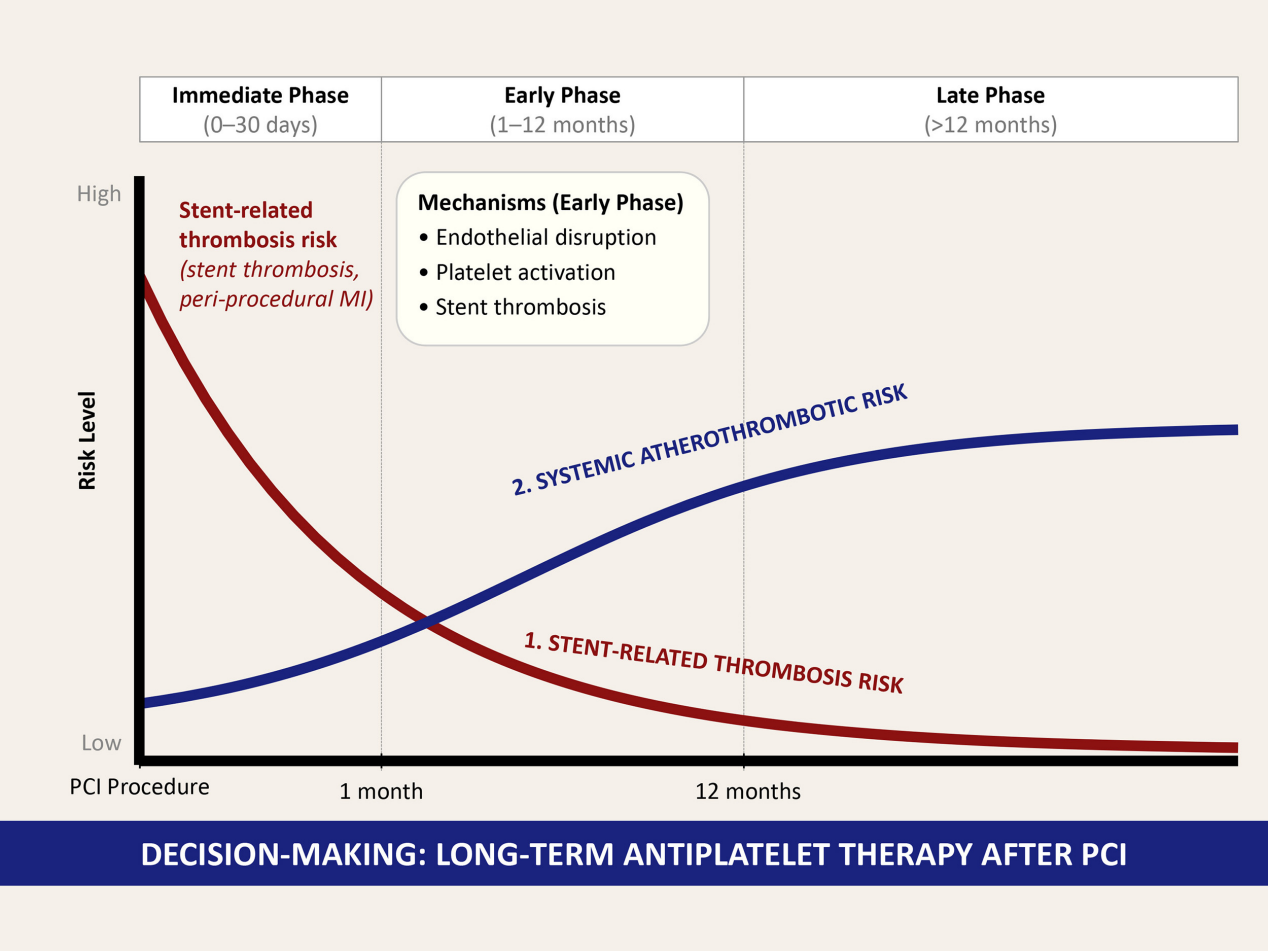
**Temporal evolution of thrombotic risk after percutaneous coronary intervention (PCI)**.

Beyond the first year after percutaneous coronary intervention (PCI), the therapeutic challenge shifts from preventing device-related thrombosis to managing systemic atherothrombotic risk. The traditional strategy of lifelong aspirin is now being called into question as trial data suggest that alternative approaches—including P2Y_12_ inhibitor monotherapy and dual pathway inhibition—may offer better protection with comparable or lower bleeding risk. Within the first 12 months, dual antiplatelet therapy (DAPT) is firmly anchored in preventing stent-related complications such as stent thrombosis, peri-procedural myocardial infarction (MI), and acute vessel occlusion [1,2,3]. As stent endothelialisation progresses, however, these device-related hazards decline, and late adverse events increasingly reflect progressive atherosclerosis, non-culprit lesion progression, and systemic vascular disease [4,5] as illustrated in Fig. 1. The clinical priority therefore moves from stent protection to broad-based secondary prevention across the coronary, cerebral, and peripheral vascular beds.

This schematic illustrates the shift in dominant thrombotic mechanisms following PCI. During the immediate phase (0–30 days), thrombotic risk is driven predominantly by stent-related factors including endothelial disruption, platelet activation, peri-procedural myocardial infarction, and incomplete endothelialisation. As vascular healing progresses through the early phase (1–12 months), the risk of stent thrombosis declines substantially. Beyond 12 months, the dominant mechanism of recurrent events becomes systemic atherothrombosis, reflecting progression of underlying coronary and polyvascular disease rather than device-related complications. This temporal transition underpins current strategies in which dual antiplatelet therapy is prioritised early after PCI, followed by individualised long-term antithrombotic therapy tailored to the individual patient’s ischaemic and bleeding risk profile.

This temporal evolution creates an important clinical decision point at 12 months. Clinicians must weigh the potential benefit of continued antithrombotic intensity against the cumulative hazard of bleeding—which itself is independently associated with mortality, recurrent ischaemic events and impaired quality of life [6,7]. The traditional default strategy of lifelong low-dose aspirin is now being challenged by evidence that alternative approaches may offer greater ischaemic protection, reduced bleeding, or both, in selected patients.

The pharmacology of aspirin and P2Y_12_ inhibitors, and the evidence for abbreviated DAPT strategies within the first 12 months after PCI (including TWILIGHT, MASTER-DAPT, STOPDAPT-2/3, NEO-MINDSET, ULTIMATE-DAPT and T-PASS) are covered in a companion review. The present article focuses on the outcomes evidence for long-term therapy beyond one year, precision-based strategies, and practical clinical decision-making at and after the 12-month transition point.

### Search Strategy and Study Selection

This narrative review draws on a structured literature search of PubMed/MEDLINE, Embase, and ClinicalTrials.gov, covering landmark dual antiplatelet therapy studies through April 2026. Search terms combined “PCI” or “percutaneous coronary intervention” with “dual antiplatelet therapy”, “P2Y_12_ inhibitor monotherapy”, “dual pathway inhibition”, “secondary prevention”, “rivaroxaban”, and the names of major trials including HOST-EXAM, PEGASUS, and COMPASS. Bleeding-risk literature was identified through the additional terms “bleeding risk”, “PRECISE-DAPT”, “Academic Research Consortium for High Bleeding Risk (ARC-HBR)”, and “PRECISE-HBR”. Priority was given to randomised controlled trials, individual patient data meta-analyses, contemporary guideline documents, and consensus statements. Observational studies were included selectively where they addressed populations under-represented in randomised evidence. This article represents a narrative synthesis rather than a systematic review; we did not apply formal Preferred Reporting Items for Systematic Reviews and Meta-Analyses (PRISMA)-based study selection or quantitative pooling, and the article should be interpreted accordingly.

## 2. Transition From DAPT to Maintenance Therapy

### 2.1 Historical Context

The adoption of 12 months as a standard DAPT duration was driven primarily by the CURE and CREDO programmes, which established that prolonged clopidogrel plus aspirin reduced ischaemic events after acute coronary syndrome (ACS) and PCI [1,2]. Concerns regarding late stent thrombosis with first-generation drug-eluting stents (DES)—particularly sirolimus- and paclitaxel-eluting platforms—prompted regulatory and guideline bodies to recommend extended dual therapy as a precautionary measure [8,9]. The progressive improvement in stent design, with thinner struts, biocompatible or biodegradable polymers, and refined drug elution kinetics in contemporary DES, has substantially reduced the incidence of very late stent thrombosis [10] and called into question the necessity of prolonged dual therapy in unselected patients.

### 2.2 Residual Risk After DAPT Completion

Patients who complete DAPT without incident retain substantial residual cardiovascular risk. Registry data from the REACH line and Swedish nationwide cohorts demonstrate that beyond one year, the annual rate of MI, stroke, or cardiovascular death remains in the order of 4–5% even in event-free survivors [11,12]. This residual risk is driven by non-culprit coronary disease progression, systemic atherosclerosis, and persistent prothrombotic factors including platelet hyperreactivity, endothelial dysfunction, and chronic inflammation. The question at the 12-month transition is not whether secondary prevention should continue, but which agent—or combination—provides the most favourable balance between ischaemic protection and bleeding risk.

## 3. Aspirin as the Traditional Default

For over three decades, low-dose aspirin has been the uncontested default for long-term secondary prevention after DAPT completion—a position now increasingly questioned by head-to-head comparative data. The Antithrombotic Trialists’ Collaboration meta-analysis demonstrated that aspirin reduced the annual risk of serious vascular events by approximately one quarter compared with placebo in patients with prior vascular events, with absolute benefits substantially outweighing excess bleeding in most secondary prevention populations [13]. Its dominance reflected not only efficacy, but also cost, familiarity, once-daily dosing, and the absence for many years of compelling long-term alternatives tested head-to-head against aspirin.

The comparator in these landmark analyses, however, was placebo or no antiplatelet therapy – not an alternative antiplatelet agent. The question of whether aspirin represents the optimal long-term agent was first raised by CAPRIE, which showed that clopidogrel 75 mg was marginally but significantly more effective than aspirin 325 mg in reducing the composite of vascular death, MI, or ischaemic stroke (annual event rate 5.32% vs 5.83%; relative risk reduction 8.7%, *p* = 0.043) [14]. Although the absolute difference was modest, CAPRIE established the principle that adenosine diphosphate (ADP)-pathway inhibition could provide at least equivalent, and perhaps modestly superior, long-term vascular protection compared with aspirin. The clinical relevance of this observation has been substantially amplified by subsequent evidence.

## 4. P2Y_12_ Inhibitor Monotherapy Beyond One Year

### 4.1 HOST-EXAM and HOST-EXAM Extended

The HOST-EXAM trial randomised 5438 patients who had completed 6–18 months of event-free DAPT after DES implantation to clopidogrel 75 mg or aspirin 100 mg monotherapy [15]. At 24 months, clopidogrel significantly reduced the primary composite of all-cause death, MI, stroke, readmission for ACS, and Bleeding Academic Research Consortium (BARC) 3–5 bleeding compared with aspirin (5.7% vs 7.7%; hazard ratio [HR] 0.73, 95% confidence interval [CI] 0.59–0.90, *p* = 0.0035). Both ischaemic and bleeding endpoints were individually lower in the clopidogrel arm—an unusual finding, since most antithrombotic interventions trade ischaemic benefit for bleeding risk.

HOST-EXAM Extended reported outcomes at a median follow-up of 5.8 years [16]. The benefit of clopidogrel was sustained throughout, with significantly lower rates of the primary composite, the secondary thrombotic endpoint, and the secondary bleeding endpoint. Landmark analysis at two years confirmed that this advantage persisted in the extended phase, with no divergence in all-cause mortality. Post-hoc analyses demonstrated consistency across strata of baseline ischaemic and bleeding risk. These data provide the strongest direct evidence to date that clopidogrel monotherapy may be preferable to aspirin for long-term maintenance after PCI. The trial population was predominantly Korean, however, and pharmacogenomic as well as broader population-level differences warrant caution when extrapolating these findings to more diverse cohorts.

More recently, the 10-year follow-up of HOST-EXAM has been reported [17]. Over a median follow-up of 10.5 years, the relative benefit of clopidogrel over aspirin monotherapy remained directionally consistent for the primary composite endpoint (25.4% vs 28.5%) and for thrombotic events (17.3% vs 20.0%), with a sustained reduction in bleeding complications (9.1% vs 10.8%). All-cause mortality did not differ significantly between the two groups. These long-term findings strengthen the biological plausibility of P2Y_12_ inhibitor monotherapy as a durable maintenance strategy, although the predominantly East Asian source population continues to constrain direct generalisability.

HOST-EXAM was conducted in a predominantly East Asian population, in which the *CYP2C19* loss-of-function allele prevalence is substantially higher than in European cohorts; the implications of this for direct generalisability to other populations remain a matter of active debate. The enrolment criterion of 6 to 18 months of event-free dual antiplatelet therapy also excluded the early high-risk peri-procedural window, which limits direct extrapolation to patients entering chronic maintenance from a more recent ischaemic event. The reduction in BARC 3–5 bleeding was modest in absolute terms but consistent in direction across pre-specified subgroups, and the trial did not demonstrate a divergence in all-cause mortality between the two monotherapies, a point that informs how the result should be weighed against alternative long-term strategies.

### 4.2 SMART-CHOICE 3

SMART-CHOICE 3 randomised 5506 high-risk patients who had completed DAPT after PCI to clopidogrel or aspirin monotherapy for three years [18]. Clopidogrel reduced the primary composite of death, MI and stroke (HR 0.71, 95% CI 0.54–0.93) with comparable rates of major bleeding. Clopidogrel was also associated with fewer gastrointestinal events—a finding consistent with the known gastrointestinal toxicity of aspirin but nonetheless reassuring in the context of monotherapy selection. Like HOST-EXAM, SMART-CHOICE 3 was conducted predominantly in Korean centres, and similar limitations apply when considering generalisability across ethnically diverse populations.

### 4.3 PANTHER Meta-Analysis

The PANTHER meta-analysis pooled individual participant data from seven randomised trials (24,325 participants) comparing P2Y_12_ inhibitor monotherapy with aspirin monotherapy for secondary prevention in patients with established coronary artery disease [19]. The primary composite of cardiovascular death, MI and stroke was significantly lower in the P2Y_12_ inhibitor group (HR 0.88, 95% CI 0.79–0.97, *p* = 0.012), driven predominantly by fewer myocardial infarctions (HR 0.77, 95% CI 0.66–0.90), with no excess of major bleeding. The magnitude of benefit was broadly consistent regardless of the P2Y_12_ agent used (predominantly clopidogrel). A subsequent individual patient data meta-analysis of five of these trials (16,117 participants) by Giacoppo et al. [20] confirmed these findings (HR 0.84, 95% CI 0.74–0.96 for the primary composite), with consistent benefit across pre-specified subgroups. A further updated analysis by Valgimigli et al. [21], incorporating 28,982 patients from seven trials with a median follow-up of 2.3 years, confirmed that clopidogrel was associated with fewer major adverse cardiovascular events than aspirin at 5.5 years, without excess mortality or major bleeding. Taken together, these data provide the most comprehensive evidence to date that P2Y_12_ inhibitor monotherapy is at least a credible and possibly preferable alternative to aspirin for long-term secondary prevention after DAPT completion.

Taken together, HOST-EXAM, HOST-EXAM Extended, SMART-CHOICE 3, and PANTHER point in the same direction: ADP-pathway inhibition appears to provide more effective long-term protection against recurrent coronary events than thromboxane inhibition alone, without an obvious bleeding penalty. Clopidogrel monotherapy therefore, merits serious consideration as a compelling alternative to aspirin as maintenance strategy after DAPT completion. This is despite the important caveat that the trial populations have been predominantly East Asian, and whether the same magnitude of benefit holds in ethnically diverse cohorts has yet to be established.

### 4.4 Pharmacogenomic Considerations

These findings must be interpreted in the context of pharmacogenomic variability in clopidogrel metabolism. The CYP2C19*2 loss-of-function allele frequency is approximately 15–18% in European populations, but the proportion of individuals carrying at least one loss-of-function allele (intermediate and poor metabolisers combined) is closer to 25–30%; the corresponding carrier frequency in East Asian populations is 30–40%, with considerable intra-regional heterogeneity [22]. These alleles reduce clopidogrel bioactivation and may attenuate clinical benefit. This is particularly relevant for long-term monotherapy, where suboptimal platelet inhibition sustained over years could translate into excess thrombotic events. The predominantly East Asian composition of HOST-EXAM and SMART-CHOICE 3 introduces an important interpretive complexity: although loss-of-function allele prevalence is high in these populations, clopidogrel still outperformed aspirin, likely reflecting a combination of pharmacogenomic, bleeding-risk, and population-specific factors. Validation in ethnically diverse cohorts remains an important unmet need.

## 5. Prolonged Dual Antiplatelet Therapy

Although the overall trajectory of evidence favours de-escalation to monotherapy beyond one year, a subset of patients with persistently high ischaemic risk may still derive net benefit from prolonged DAPT.

### 5.1 DAPT Trial

The DAPT Trial randomised 9961 patients who had completed 12 months of uneventful DAPT after DES implantation to continued dual therapy for an additional 18 months or placebo plus aspirin [3]. Continued DAPT significantly reduced stent thrombosis (0.4% vs 1.4%, *p* < 0.001) and spontaneous MI (2.1% vs 4.1%, *p* < 0.001), but increased moderate-to-severe bleeding (2.5% vs 1.6%, *p* = 0.001) and was associated with a numerical excess of non-cardiovascular death. These findings crystallised the central trade-off: reduction in ischaemic events comes at a measurable haemorrhagic cost, and the net balance depends on individual patient risk rather than a uniform timeline.

These findings position prolonged dual antiplatelet therapy most clearly in patients with persistently elevated ischaemic risk and acceptable bleeding liability. The absolute reductions in stent thrombosis and spontaneous myocardial infarction must be weighed against the absolute increase in moderate-to-severe bleeding, and a numerically higher rate of non-cardiovascular mortality observed in the extended-treatment arm has remained the subject of continued debate. The applicability of these data to contemporary thin-strut drug-eluting platforms is also uncertain, given that newer stents themselves carry a lower late stent-thrombosis hazard than the devices in the DAPT Trial.

### 5.2 PEGASUS–TIMI 54

PEGASUS–Thrombolysis in Myocardial Infarction (TIMI) 54 enrolled 21,162 patients with a history of MI one to three years earlier and randomised them to ticagrelor 60 mg or 90 mg twice daily plus aspirin, or aspirin alone [23]. Over a median of 33 months, ticagrelor 60 mg significantly reduced the composite of cardiovascular death, MI or stroke (HR 0.84, 95% CI 0.74–0.95, *p* = 0.004), driven largely by fewer MIs. TIMI major bleeding was increased, though intracranial and fatal bleeding remained infrequent. The 60 mg dose provided similar ischaemic benefit to the 90 mg dose with a more favourable tolerability profile and is the preferred regimen.

The PEGASUS-TIMI 54 finding applies most directly to patients with a prior myocardial infarction one to three years earlier and acceptable bleeding risk. The 60 mg twice-daily dose was preferred over 90 mg on the basis of comparable ischaemic efficacy with a modestly more favourable bleeding profile, and intracranial and fatal bleeding events were uncommon in absolute terms. The trial illustrates the more general principle that absolute ischaemic benefit and absolute bleeding harm tend to move together with treatment intensification, and that the magnitude of each must be weighed against the patient’s baseline risk profile.

### 5.3 Patient Selection

Prolonged DAPT is best reserved for patients with high ischaemic burden and low bleeding risk. Clinical scenarios in which this approach may be considered include complex left main or bifurcation stenting, prior stent thrombosis, extensive multivessel disease with incomplete revascularisation, recurrent MI, and the combination of diabetes with diffuse coronary disease. The DAPT score, applied at 12 months, helps discriminate patients predicted to derive net benefit (score ≥2) from those more likely to experience net harm (score <2) [24].

The message from both the DAPT Trial and PEGASUS–TIMI 54 is consistent: prolonged dual therapy reduces stent thrombosis and spontaneous MI, but at a real haemorrhagic cost. Net benefit is therefore confined to the subset of patients whose ischaemic burden clearly outweighs their bleeding vulnerability—and identifying that subset requires structured risk assessment at 12 months.

## 6. Dual Pathway Inhibition

Recognition that atherothrombosis is driven by both platelet activation and thrombin generation has prompted evaluation of dual pathway inhibition—combining antiplatelet therapy with low-dose anticoagulation.

### 6.1 COMPASS

COMPASS randomised 27,395 patients with stable atherosclerotic vascular disease to rivaroxaban 2.5 mg twice daily plus aspirin, rivaroxaban 5 mg twice daily alone, or aspirin alone [25]. The combination significantly reduced the composite of cardiovascular death, stroke, or MI compared with aspirin alone (4.1% vs 5.4%; HR 0.76, 95% CI 0.66–0.86, *p* < 0.001) and yielded a favourable net clinical benefit despite increased major bleeding. Critically, fatal and intracranial bleeding did not increase significantly. Subgroup analyses confirmed that the greatest net benefit was in patients with polyvascular disease, impaired renal function, heart failure and diabetes [26]. Although COMPASS was not restricted to post-PCI patients, a substantial proportion had prior coronary revascularisation, supporting extrapolation to stable post-PCI populations with high residual risk.

COMPASS established dual pathway inhibition as a viable long-term strategy in stable atherosclerotic disease and is notable as one of the few contemporary regimens to demonstrate a reduction in all-cause mortality alongside the ischaemic composite. The treatment effect was driven disproportionately by patients with polyvascular involvement, and the trial excluded those at the highest bleeding risk. Fatal and intracranial bleeding were not increased relative to aspirin alone, although total bleeding events were, and the absolute trade-off remains the central consideration in patient selection.

### 6.2 VOYAGER-PAD

VOYAGER-PAD demonstrated that in 6564 patients with symptomatic peripheral artery disease (PAD) undergoing revascularisation, low-dose rivaroxaban plus aspirin reduced a composite of limb and systemic ischaemic events compared with aspirin alone (17.3% vs 19.9%; HR 0.85, 95% CI 0.76–0.96, *p* = 0.009) [27]. Although this population differs from typical post-PCI cohorts, the findings reinforce the biology of dual pathway inhibition: thrombin-mediated pathways contribute materially to recurrent atherothrombosis, particularly in polyvascular disease.

### 6.3 Clinical Positioning

Dual pathway inhibition occupies a distinct therapeutic niche. It is most relevant for patients with established coronary disease and high residual vascular risk—particularly those with polyvascular disease—in whom bleeding risk is not prohibitive. The 2019 European Society of Cardiology (ESC) chronic coronary syndromes guidelines recommended considering rivaroxaban 2.5 mg twice daily plus aspirin in such patients [28]; the 2024 update has since refined the strength and scope of this recommendation [29]. An important unanswered question is whether this strategy offers incremental benefit over P2Y_12_ inhibitor monotherapy for long-term secondary prevention, since no head-to-head randomised trial has directly compared the two approaches. In practice, the choice should be guided by the individual’s vascular phenotype (single vs multiple vascular beds), bleeding risk, renal function, and co-existing indications.

COMPASS and VOYAGER-PAD together confirm that thrombin-mediated pathways contribute materially to recurrent atherothrombosis, and that targeting both platelet activation and coagulation amplification can produce clinically meaningful ischaemic reductions. The bleeding cost is real but appears manageable in the right patients—principally those with polyvascular disease or multiple co-existing risk factors.

## 7. Additional Intensification Strategies: Protease-Activated Receptor Antagonism

### 7.1 TRA 2°P–TIMI 50 (vorapaxar)

TRA 2°P–TIMI 50 evaluated vorapaxar, a first-in-class protease-activated receptor (PAR)-1 antagonist, in 26,449 patients with established atherosclerotic disease [30]. Among patients with prior MI—roughly 60% of the trial—vorapaxar added to standard antiplatelet therapy significantly reduced the composite of cardiovascular death, MI, or stroke (HR 0.80, 95% CI 0.72–0.89, *p* < 0.001). This came, however, at the cost of significantly increased GUSTO moderate-to-severe bleeding, including a higher rate of intracranial haemorrhage—particularly in patients with prior cerebrovascular disease, leading to early termination of the stroke arm. Vorapaxar was subsequently approved for secondary prevention in selected post-MI patients without prior stroke, but clinical uptake has remained limited because of bleeding concerns and the narrow qualifying population.

### 7.2 THEMIS and THEMIS-PCI

THEMIS randomised 19,220 patients with type 2 diabetes and stable coronary artery disease (without prior MI or stroke) to ticagrelor 60 mg twice daily plus aspirin or aspirin alone [31]. The primary composite was modestly reduced (HR 0.90, 95% CI 0.81–0.99, *p* = 0.04), but TIMI major bleeding was substantially increased, yielding an uncertain net benefit overall. The pre-specified THEMIS-PCI subgroup (11,154 patients with prior PCI) demonstrated a more favourable risk–benefit balance: ticagrelor significantly reduced the primary ischaemic endpoint, and net clinical benefit favoured the combination [32]. These data suggest that diabetic patients with prior PCI represent a higher-risk phenotype in whom long-term intensified antiplatelet therapy may merit consideration, though blanket adoption in all diabetic post-PCI patients cannot be recommended without individual risk assessment.

## 8. Special Populations

### 8.1 Diabetes Mellitus

Patients with diabetes exhibit persistent platelet hyperreactivity and elevated residual vascular risk [33]. Evidence from THEMIS-PCI and consistent subgroup findings from PEGASUS–TIMI 54 support the potential benefit of intensified long-term antiplatelet therapy in this group. Despite this biological rationale, evidence supporting routine prolonged DAPT in unselected diabetic populations remains inconsistent, and individualised therapy based on bleeding risk is generally preferred.

### 8.2 Chronic Kidney Disease

Chronic kidney disease (CKD) produces a paradoxical combination of bleeding tendency and thrombotic risk, compounded by altered pharmacokinetics and the exclusion of severe renal impairment from most major trials [34]. The COMPASS CKD subgroup demonstrated that patients with estimated glomerular filtration rate <60 mL/min/1.73 m^2^ derived at least as great a benefit from dual pathway inhibition as those with preserved renal function [26]. An individualised approach accounting for renal trajectory and competing risks is essential in this population.

At a mechanistic level, the bleeding-thrombosis paradox in CKD reflects two distinct pathologies operating simultaneously: reduced glomerular filtration is associated with platelet hyperreactivity and accelerated atherothrombosis, while uraemia produces platelet dysfunction, capillary fragility, and altered drug pharmacokinetics. Clopidogrel responsiveness becomes increasingly variable as renal function declines, reflecting both genetic *CYP2C19* variation and CKD-specific metabolic determinants. Patients on dialysis remain substantially underrepresented in randomised antithrombotic trials, and most current recommendations for this group rely on indirect inference from non-dialysis CKD cohorts. CKD represents one of the clearest examples of competing ischaemic and bleeding hazards coexisting simultaneously, and individualised decision-making in this population should remain explicit about the limits of the supporting evidence.

### 8.3 Polyvascular Disease

Patients with atherosclerosis affecting two or more vascular beds have substantially higher rates of recurrent events and represent the quintessential high-risk population for intensive long-term secondary prevention [35]. COMPASS demonstrated that these patients derive the greatest net benefit from rivaroxaban plus aspirin, strongly supporting dual pathway inhibition as the preferred long-term strategy where bleeding risk is acceptable.

### 8.4 High Bleeding Risk and Elderly Patients

Elderly patients, particularly those over 75 years, experience disproportionately higher rates of major bleeding on any antithrombotic regimen [36]. HOST-EXAM and SMART-CHOICE 3 both suggest that clopidogrel is at least as effective as aspirin while conferring lower gastrointestinal bleeding risk [15,18]. Long-term clopidogrel monotherapy is therefore an attractive option in elderly patients in whom bleeding is the dominant concern, especially when combined with proton pump inhibitor gastroprotection.

Beyond age itself, the elderly post-PCI population is characterised by frailty, polypharmacy, anaemia, falls risk, and prior gastrointestinal bleeding, all of which independently modify the bleeding-ischaemia balance. Altered absorption, plasma protein binding, and renal clearance further contribute to pharmacokinetic variability. The MASTER-DAPT experience supports the principle that abbreviated dual antiplatelet therapy followed by transition to monotherapy can preserve ischaemic protection while reducing bleeding events in selected high-bleeding-risk PCI patients. Chronological age alone should not determine treatment intensity, although advancing age consistently modifies both bleeding and ischaemic risk; simplified regimens are generally preferable in this population unless a specific ischaemic indication overrides the bleeding consideration.

### 8.5 Atrial Fibrillation and Oral Anticoagulation

Patients with atrial fibrillation (AF) who have undergone PCI require careful balancing of cardioembolic and coronary thrombotic risk. After the initial period of combined therapy, contemporary guidelines—informed by AFIRE and AUGUSTUS—recommend direct oral anticoagulant (DOAC) monotherapy beyond 12 months, discontinuing all antiplatelet agents unless other compelling indications exist [37,38]. Whether adding long-term P2Y_12_ monotherapy to a DOAC beyond the first year confers additional coronary benefit remains unproven and may expose patients to avoidable bleeding. In the absence of another clear indication for antiplatelet therapy, DOAC monotherapy remains the preferred long-term strategy. The shorter-triple-therapy direction has been further reinforced by PIONEER-AF-PCI and ENTRUST-AF-PCI, which support DOAC-based regimens with reduced antiplatelet exposure in patients requiring long-term anticoagulation.

### 8.6 Sex-Specific Considerations

Women remain substantially underrepresented in PCI trials, and the bleeding-ischaemia trade-off differs between sexes in ways that the current evidence base does not fully characterise. Women tend to present at older ages, with smaller-calibre vessels and a greater burden of frailty, all of which independently raise bleeding risk on any antithrombotic regimen. A recently published sex-stratified individual patient data meta-analysis has begun to address this gap [39], although definitive sex-specific therapeutic algorithms remain unavailable, and routine practice continues to rely on sex-neutral recommendations supplemented by individualised judgement.

The principal randomised trials informing long-term antithrombotic strategies after PCI are summarised in Table 1 (Ref. [3,15,18,19,23,25,27]).

**Table 1.** **Summary of key trials informing long-term antithrombotic therapy after PCI**.

Trial	Population (n)	Strategy vs comparator	Primary endpoint HR (95% CI)	Key finding
HOST-EXAM [15]	Post-DES, event-free (5438)	Clopidogrel vs aspirin	0.73 (0.59–0.90)	Clopidogrel superior for ischaemic and bleeding endpoints
SMART-CHOICE 3 [18]	High-risk post-PCI (5506)	Clopidogrel vs aspirin (3 yr)	0.71 (0.54–0.93)	Clopidogrel reduced death/MI/stroke with comparable bleeding
PANTHER (IPD MA) [19]	Established CAD (24,325)	P2Y_12_ mono vs aspirin mono	0.88 (0.79–0.97)	P2Y_12_ monotherapy favoured; driven by fewer MIs
DAPT Trial [3]	Post-DES at 12 mo (9961)	30 mo DAPT vs 12 mo DAPT	0.29 (0.17–0.48)	Reduced ST and MI; increased moderate–severe bleeding
PEGASUS–TIMI 54 [23]	Prior MI 1–3 yr (21,162)	Ticagrelor 60 mg + ASA vs ASA	0.84 (0.74–0.95)	Reduced CV death/MI/stroke; 60 mg preferred over 90 mg
COMPASS [25]	Stable ASCVD (27,395)	Rivaroxaban 2.5 mg BD + ASA vs ASA	0.76 (0.66–0.86)	Net clinical benefit; greatest in polyvascular disease
VOYAGER-PAD [27]	PAD revascularisation (6564)	Rivaroxaban 2.5 mg BD + ASA vs ASA	0.85 (0.76–0.96)	Reduced limb and systemic ischaemic events

ASA, aspirin; ASCVD, atherosclerotic cardiovascular disease; BD, twice daily; CAD, coronary artery disease; CI, confidence interval; CV, cardiovascular; DES, drug-eluting stent; HR, hazard ratio; IPD MA, individual patient data meta-analysis; MI, myocardial infarction; PAD, peripheral artery disease; PCI, percutaneous coronary intervention; ST, stent thrombosis.

## 9. Precision Strategies

### 9.1 CYP2C19 Genotype-Guided Therapy

POPular Genetics demonstrated that *CYP2C19* genotype-guided P2Y_12_ inhibitor selection was non-inferior for combined ischaemic and bleeding events and significantly reduced bleeding compared with universal use of potent P2Y_12_ inhibitors in primary PCI for ST-segment elevation myocardial infarction (STEMI) [40]. TAILOR-PCI did not meet its primary endpoint (HR 0.66, 95% CI 0.43–1.02), but a pre-specified multiple-events analysis suggested that loss-of-function carriers treated with genotype-guided therapy had fewer recurrent cardiovascular events than those on conventional clopidogrel [41]; this finding should be considered hypothesis-generating. These trials support genotype-guided selection as a plausible strategy for optimising long-term P2Y_12_ inhibitor choice, particularly when clopidogrel is being considered. Since genotype is stable, a single test performed near the index event can inform years of subsequent prescribing.

### 9.2 Platelet Function Testing

Platelet function testing directly measures pharmacodynamic effect. Although high on-treatment platelet reactivity is associated with adverse outcomes in observational studies, the ARCTIC trial and others have not shown that adjusting therapy based on platelet function testing improves hard clinical endpoints [42]. Routine use for long-term management is not currently recommended, though it may have a role in patients with recurrent events where pharmacogenomic data are unavailable.

### 9.3 Risk Scores

The DAPT score helps predict the net benefit of extending DAPT beyond 12 months [24]. The PRECISE-DAPT score, validated primarily for early post-PCI bleeding risk, may also inform long-term decisions [43]. The ARC-HBR criteria provide a standardised framework for identifying patients in whom de-escalation should be prioritised [44]. Machine-learning models incorporating clinical, imaging, and biomarker data are under development but remain exploratory and are not yet ready to inform routine post-PCI prescribing.

The recently published PRECISE-HBR score offers a more contemporary approach to bleeding-risk stratification after PCI by integrating elements of PRECISE-DAPT and the ARC-HBR criteria within a single seven-item instrument [45]. Derived from 29,188 patients undergoing PCI and externally validated in two additional cohorts totalling 10,548 patients, the score incorporates age, renal function, haemoglobin, white-cell count, prior bleeding, oral anticoagulation use, and the formal ARC-HBR criteria. External validation showed modest discrimination improvement over existing scores, with an area under the curve of 0.73 to 0.74. PRECISE-HBR may offer incremental refinement of bleeding-risk assessment in routine practice, although prospective implementation studies are still awaited. Bleeding risk is dynamic and evolves over time; risk scores should complement rather than replace clinical judgement.

## 10. Future Directions

Several therapeutic avenues may yet refine the long-term antithrombotic approach after PCI, although recent experience has been mixed.

### 10.1 Factor XIa Inhibitors

Factor XIa inhibitors target the amplification loop of coagulation while largely sparing primary haemostasis [46]. Genetic data suggest that factor XI deficiency confers thrombotic protection with modest bleeding. Data from OCEANIC-STROKE suggested a possible reduction in recurrent ischaemic stroke with a reassuring bleeding profile [47]. However, OCEANIC-AF was stopped early because asundexian was inferior to apixaban for stroke prevention in AF [48], and the phase III LIBREXIA-ACS trial of milvexian was terminated in 2025 after a pre-planned interim analysis indicating a low probability of demonstrating clinical benefit [49]. These mixed results suggest that factor XIa inhibition may prove indication-specific rather than broadly transferable across vascular syndromes, and its role in long-term post-PCI management remains to be defined.

### 10.2 PAR-4 Antagonists and Other Novel Agents

BMS-986120, an oral PAR-4 antagonist, demonstrated potent, selective, and reversible inhibition of PAR-4–mediated platelet activation in phase I studies, with favourable effects on *ex vivo* thrombus formation under high shear [50]. By targeting thrombin-mediated platelet activation while preserving other haemostatic pathways, PAR-4 antagonism may reduce thrombotic events without a proportional increase in bleeding. Selatogrel, a subcutaneous P2Y_12_ antagonist with rapid onset, is being evaluated primarily for pre-hospital ACS [51], but its pharmacology illustrates the broader trend towards flexible, rapidly deployable antiplatelet strategies. Intravascular imaging-guided PCI—using optical coherence tomography to ensure optimal stent deployment—may also permit more confident DAPT abbreviation [52], though whether this translates into altered long-term antithrombotic requirements remains to be demonstrated.

### 10.3 Glycoprotein VI Inhibition

Glycoprotein VI (GPVI) mediates platelet-collagen interaction at sites of vascular injury and represents a mechanistically attractive target for selectively reducing pathological thrombosis while preserving physiological haemostasis. Revacept, a recombinant soluble dimeric GPVI-Fc fusion protein, was evaluated in elective PCI in the ISAR-PLASTER trial, where it did not reduce the composite of death or myocardial injury compared with placebo (24.4% versus 23.3% at 30 days) but produced no excess of Bleeding Academic Research Consortium type 2 or higher bleeding [53]. Glenzocimab, a humanised antigen-binding fragment targeting platelet GPVI, has been studied predominantly outside the PCI setting, including in acute ischaemic stroke (ACTISAVE, ongoing) and in COVID-19, where it has shown a favourable safety profile without inducing haemorrhage [54]. Clinical outcome data in PCI populations remain limited, and the role of GPVI inhibition in long-term post-PCI care is not yet defined.

### 10.4 Colchicine

Although colchicine is primarily an anti-inflammatory agent, experimental data suggest modest indirect interactions with thrombotic pathways, including attenuation of platelet-leukocyte signalling. The proof-of-concept MACT pilot trial (n = 200 patients with acute coronary syndromes undergoing PCI) reported that aspirin discontinuation with continuation of ticagrelor or prasugrel monotherapy alongside low-dose colchicine appeared feasible, with stent thrombosis in 1.0% at three months [55]. The trial was a pilot study not powered for clinical efficacy outcomes, and the findings should be regarded as hypothesis-generating; aspirin replacement in this setting is not validated by these data.

### 10.5 Icosapent Ethyl

Recent mechanistic data suggest that icosapent ethyl may exert direct platelet-modulating effects via cyclooxygenase-1 (COX-1)–related pathways, in addition to its broader anti-inflammatory and plaque-stabilising properties [56]. Although icosapent ethyl is not an antithrombotic therapy in its own right, these effects may contribute to cardiovascular risk reduction in selected high-risk patients receiving standard antiplatelet regimens.

## 11. Clinical Decision Framework

**Fig. 2.**
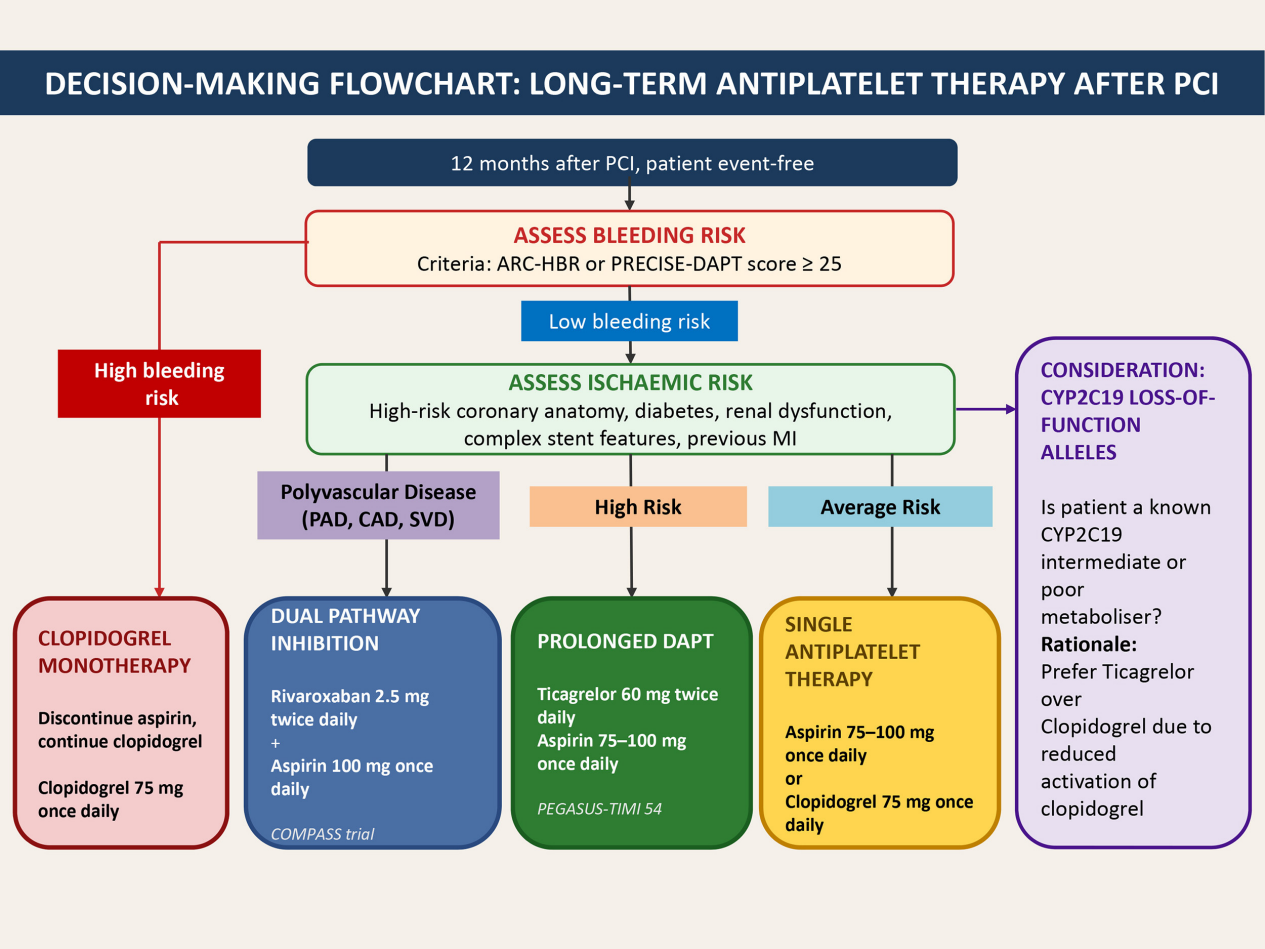
**Clinical decision framework for long-term antiplatelet therapy after PCI**.

The therapeutic options outlined above require translation into a practical framework at the 12-month transition point, where the central question is no longer whether treatment should continue, but how it should be individualised. A practical approach to antithrombotic strategy at 12 months should integrate ischaemic risk, bleeding risk, vascular phenotype, pharmacogenomic status, and patient preference as shown in Table 2 and a simplified clinical decision algorithm is shown in Fig. 2. This table also summarises the relationship between dominant risk phenotype and the most appropriate long-term therapeutic strategy.

**Table 2.** **Evidence-mapped clinical decision framework for long-term antithrombotic therapy at and beyond 12 months after PCI**.

Clinical scenario	Dominant risk phenotype	Recommended strategy	Key supporting evidence
High bleeding risk (ARC-HBR/PRECISE-DAPT ≥25) or elderly (≥75 years)	Bleeding-dominant	Single antiplatelet therapy; clopidogrel preferred over aspirin	*HOST-EXAM*, *PANTHER*, *SMART-CHOICE 3*
Average risk, event-free at 12 months	Balanced	P2Y_12_ inhibitor monotherapy (clopidogrel preferred); consider *CYP2C19* status if available	*HOST-EXAM*, *PANTHER*, *CAPRIE*
High ischaemic risk, low bleeding risk (prior MI, complex PCI, diffuse CAD)	Ischaemia-dominant	Prolonged DAPT (ticagrelor 60 mg + aspirin) or dual pathway inhibition (rivaroxaban 2.5 mg BD + aspirin)	*PEGASUS–TIMI 54*, *DAPT Trial*, *COMPASS*
Polyvascular disease (coronary + peripheral or cerebrovascular)	Ischaemia-dominant	Dual pathway inhibition: rivaroxaban 2.5 mg BD + aspirin	*COMPASS*, *VOYAGER-PAD*
Diabetes with stable CAD and prior PCI	Ischaemia-dominant	Consider ticagrelor 60 mg + aspirin if bleeding risk acceptable; dual pathway inhibition an alternative	*THEMIS-PCI*, *PEGASUS–TIMI 54*, *COMPASS*
*CYP2C19* loss-of-function carrier on clopidogrel	Variable	Consider ticagrelor or prasugrel; revert to aspirin if bleeding risk high and potent P2Y_12_ inhibitor not tolerated	*POPular Genetics*, *TAILOR-PCI*
AF requiring long-term oral anticoagulation	Bleeding-dominant	DOAC monotherapy; routine antiplatelet addition not recommended beyond initial combined period	*AFIRE*, *AUGUSTUS*

AF, atrial fibrillation; ARC-HBR, Academic Research Consortium for High Bleeding Risk; BD, twice daily; CAD, coronary artery disease; DAPT, dual antiplatelet therapy; DOAC, direct oral anticoagulant; MI, myocardial infarction; PCI, percutaneous coronary intervention.

Proposed algorithm for antithrombotic management in patients who remain event-free 12 months after PCI. The first step is assessment of bleeding risk using established tools such as ARC-HBR or PRECISE-DAPT. In patients with high bleeding risk, single antiplatelet therapy is preferred, with clopidogrel monotherapy often favoured over aspirin. In patients with low bleeding risk, subsequent assessment of ischaemic risk guides therapy selection. Patients with polyvascular disease may benefit from dual pathway inhibition with low-dose rivaroxaban plus aspirin, while those with high ischaemic risk may be considered for prolonged dual antiplatelet therapy with ticagrelor plus aspirin. Patients at average risk may continue single antiplatelet therapy. Pharmacogenomic considerations, including *CYP2C19* loss-of-function alleles, may influence the choice of P2Y_12_ inhibitor.

This framework should be applied flexibly, with regular reassessment as the clinical trajectory evolves. Shared decision-making—incorporating patient preferences regarding tablet burden, twice-daily dosing, and risk tolerance—is essential.

The proposed framework should be interpreted as a practical synthesis of contemporary evidence rather than a definitive treatment algorithm. Direct head-to-head comparisons between long-term strategies remain scarce, several pivotal trials enrolled predominantly East Asian or European populations, and bleeding definitions vary across studies in ways that complicate quantitative comparison. Absolute event rates in modern PCI populations are lower than in historical cohorts, and the evidence base continues to evolve. Risk scores complement but do not replace clinical judgement, and the framework should be reassessed against new trial evidence as it accumulates.

## 12. Conclusions

The optimal long-term antithrombotic strategy after PCI is increasingly defined not by rigid timelines but by evolving ischaemic and bleeding risk. The 12-month mark represents a critical juncture at which clinicians should reassess the balance between residual ischaemic risk and cumulative bleeding hazard.

Lifelong aspirin—the traditional default—is now challenged by growing evidence that P2Y_12_ inhibitor monotherapy, particularly clopidogrel, may provide at least comparable and in some settings superior cardiovascular protection, without an apparent increase in major bleeding. HOST-EXAM, HOST-EXAM Extended, SMART-CHOICE 3, and PANTHER converge on this conclusion, though predominantly from East Asian populations. For patients at high ischaemic risk, prolonged DAPT with low-dose ticagrelor (PEGASUS–TIMI 54) or dual pathway inhibition with low-dose rivaroxaban (COMPASS) offer evidence-based alternatives, each with distinct advantages and trade-offs. Protease-activated receptor antagonism provides additional ischaemic protection in selected post-MI patients but at the expense of increased bleeding (TRA 2°P–TIMI 50), and intensified antiplatelet therapy may be warranted in diabetic patients with prior PCI (THEMIS-PCI).

Precision strategies—including *CYP2C19* genotyping and structured risk scores—provide tools to individualise these decisions. Novel agents targeting factor XIa and PAR-4 are under active investigation but face an uneven development path, with recent trial failures tempering initial enthusiasm. The question is not whether lifelong aspirin should remain the default after PCI—increasingly, the evidence challenges aspirin as the default—but which alternative best fits the individual patient. For most, P2Y_12_ inhibitor monotherapy offers a favourable balance of protection and safety; for those with high ischaemic burden, intensified strategies are warranted; and for those with elevated bleeding risk, a simplified regimen guided by structured assessment remains the prudent course.

## Abbreviations

ACS, acute coronary syndrome; AF, atrial fibrillation; ARC-HBR, Academic Research Consortium for High Bleeding Risk; BARC, Bleeding Academic Research Consortium; CAD, coronary artery disease; CI, confidence interval; CKD, chronic kidney disease; *CYP*, cytochrome P450; DAPT, dual antiplatelet therapy; DES, drug-eluting stent; DOAC, direct oral anticoagulant; ESC, European Society of Cardiology; HR, hazard ratio; MI, myocardial infarction; PAD, peripheral artery disease; PAR, protease-activated receptor; PCI, percutaneous coronary intervention; STEMI, ST-segment elevation myocardial infarction; TIMI, Thrombolysis in Myocardial Infarction.

## References

[1] Yusuf S, Zhao F, Mehta SR, Chrolavicius S, Tognoni G, Fox KK (2001). Effects of clopidogrel in addition to aspirin in patients with acute coronary syndromes without ST-segment elevation. The New England Journal of Medicine.

[2] Steinhubl SR, Berger PB, Mann JT, Fry ETA, DeLago A, Wilmer C (2002). Early and sustained dual oral antiplatelet therapy following percutaneous coronary intervention: a randomized controlled trial. JAMA.

[3] Mauri L, Kereiakes DJ, Yeh RW, Driscoll-Shempp P, Cutlip DE, Steg PG (2014). Twelve or 30 months of dual antiplatelet therapy after drug-eluting stents. The New England Journal of Medicine.

[4] Collet JP, Thiele H, Barbato E, Barthélémy O, Bauersachs J, Bhatt DL (2021). 2020 ESC Guidelines for the management of acute coronary syndromes in patients presenting without persistent ST-segment elevation. European Heart Journal.

[5] Stone GW, Maehara A, Lansky AJ, de Bruyne B, Cristea E, Mintz GS (2011). A prospective natural-history study of coronary atherosclerosis. The New England Journal of Medicine.

[6] Mehran R, Baber U, Steg PG, Ariti C, Weisz G, Witzenbichler B (2013). Cessation of dual antiplatelet treatment and cardiac events after percutaneous coronary intervention (PARIS): 2 year results from a prospective observational study. Lancet (London, England).

[7] Généreux P, Giustino G, Witzenbichler B, Weisz G, Stuckey TD, Rinaldi MJ (2015). Incidence, Predictors, and Impact of Post-Discharge Bleeding After Percutaneous Coronary Intervention. Journal of the American College of Cardiology.

[8] Camenzind E, Steg PG, Wijns W (2007). Stent thrombosis late after implantation of first-generation drug-eluting stents: a cause for concern. Circulation.

[9] Pfisterer M, Brunner-La Rocca HP, Buser PT, Rickenbacher P, Hunziker P, Mueller C (2006). Late clinical events after clopidogrel discontinuation may limit the benefit of drug-eluting stents: an observational study of drug-eluting versus bare-metal stents. Journal of the American College of Cardiology.

[10] Palmerini T, Biondi-Zoccai G, Della Riva D, Mariani A, Sabaté M, Smits PC (2014). Clinical outcomes with bioabsorbable polymer- versus durable polymer-based drug-eluting and bare-metal stents: evidence from a comprehensive network meta-analysis. Journal of the American College of Cardiology.

[11] Bhatt DL, Eagle KA, Ohman EM, Hirsch AT, Goto S, Mahoney EM (2010). Comparative determinants of 4-year cardiovascular event rates in stable outpatients at risk of or with atherothrombosis. JAMA.

[12] Jernberg T, Hasvold P, Henriksson M, Hjelm H, Thuresson M, Janzon M (2015). Cardiovascular risk in post-myocardial infarction patients: nationwide real world data demonstrate the importance of a long-term perspective. European Heart Journal.

[13] Antithrombotic Trialists' Collaboration (2002). Collaborative meta-analysis of randomised trials of antiplatelet therapy for prevention of death, myocardial infarction, and stroke in high risk patients. BMJ (Clinical Research Ed.).

[14] CAPRIE Steering Committee (1996). A randomised, blinded, trial of clopidogrel versus aspirin in patients at risk of ischaemic events (CAPRIE). CAPRIE Steering Committee. Lancet (London, England).

[15] Koo BK, Kang J, Park KW, Rhee TM, Yang HM, Won KB (2021). Aspirin versus clopidogrel for chronic maintenance monotherapy after percutaneous coronary intervention (HOST-EXAM): an investigator-initiated, prospective, randomised, open-label, multicentre trial. Lancet (London, England).

[16] Kang J, Park KW, Lee H, Hwang D, Yang HM, Rha SW (2023). Aspirin Versus Clopidogrel for Long-Term Maintenance Monotherapy After Percutaneous Coronary Intervention: The HOST-EXAM Extended Study. Circulation.

[17] Kang J, Park S, Yang HM, Shin ES, Rha SW, Bae JW (2026). Aspirin versus clopidogrel for chronic maintenance monotherapy after percutaneous coronary intervention: 10-year follow-up of the HOST-EXAM trial. Lancet (London, England).

[18] Choi KH, Park YH, Lee JY, Jeong JO, Kim CJ, Yun KH (2025). Efficacy and safety of clopidogrel versus aspirin monotherapy in patients at high risk of subsequent cardiovascular event after percutaneous coronary intervention (SMART-CHOICE 3): a randomised, open-label, multicentre trial. Lancet (London, England).

[19] Gragnano F, Cao D, Pirondini L, Franzone A, Kim HS, von Scheidt M (2023). P2Y_12_ Inhibitor or Aspirin Monotherapy for Secondary Prevention of Coronary Events. Journal of the American College of Cardiology.

[20] Giacoppo D, Gragnano F, Watanabe H, Kimura T, Kang J, Park KW (2025). P2Y_12_ inhibitor or aspirin after percutaneous coronary intervention: individual patient data meta-analysis of randomised clinical trials. BMJ (Clinical Research Ed.).

[21] Valgimigli M, Choi KH, Giacoppo D, Gragnano F, Kimura T, Watanabe H (2025). Clopidogrel versus aspirin for secondary prevention of coronary artery disease: a systematic review and individual patient data meta-analysis. Lancet (London, England).

[22] Scott SA, Sangkuhl K, Stein CM, Hulot JS, Mega JL, Roden DM (2013). Clinical Pharmacogenetics Implementation Consortium guidelines for CYP2C19 genotype and clopidogrel therapy: 2013 update. Clinical Pharmacology and Therapeutics.

[23] Bonaca MP, Bhatt DL, Cohen M, Steg PG, Storey RF, Jensen EC (2015). Long-term use of ticagrelor in patients with prior myocardial infarction. The New England Journal of Medicine.

[24] Yeh RW, Secemsky EA, Kereiakes DJ, Normand SLT, Gershlick AH, Cohen DJ (2016). Development and Validation of a Prediction Rule for Benefit and Harm of Dual Antiplatelet Therapy Beyond 1 Year After Percutaneous Coronary Intervention. JAMA.

[25] Eikelboom JW, Connolly SJ, Bosch J, Dagenais GR, Hart RG, Shestakovska O (2017). Rivaroxaban with or without Aspirin in Stable Cardiovascular Disease. The New England Journal of Medicine.

[26] Anand SS, Eikelboom JW, Dyal L, Bosch J, Neumann C, Widimsky P (2019). Rivaroxaban Plus Aspirin Versus Aspirin in Relation to Vascular Risk in the COMPASS Trial. Journal of the American College of Cardiology.

[27] Bonaca MP, Bauersachs RM, Anand SS, Debus ES, Nehler MR, Patel MR (2020). Rivaroxaban in Peripheral Artery Disease after Revascularization. The New England Journal of Medicine.

[28] Knuuti J, Wijns W, Saraste A, Capodanno D, Barbato E, Funck-Brentano C (2020). 2019 ESC Guidelines for the diagnosis and management of chronic coronary syndromes. European Heart Journal.

[29] Vrints C, Andreotti F, Koskinas KC, Rossello X, Adamo M, Ainslie J (2024). 2024 ESC Guidelines for the management of chronic coronary syndromes. European Heart Journal.

[30] Morrow DA, Braunwald E, Bonaca MP, Ameriso SF, Dalby AJ, Fish MP (2012). Vorapaxar in the secondary prevention of atherothrombotic events. The New England Journal of Medicine.

[31] Steg PG, Bhatt DL, Simon T, Fox K, Mehta SR, Harrington RA (2019). Ticagrelor in Patients with Stable Coronary Disease and Diabetes. The New England Journal of Medicine.

[32] Bhatt DL, Steg PG, Mehta SR, Leiter LA, Simon T, Fox K (2019). Ticagrelor in patients with diabetes and stable coronary artery disease with a history of previous percutaneous coronary intervention (THEMIS-PCI): a phase 3, placebo-controlled, randomised trial. Lancet (London, England).

[33] Ferreiro JL, Angiolillo DJ (2011). Diabetes and antiplatelet therapy in acute coronary syndrome. Circulation.

[34] Lutz J, Menke J, Sollinger D, Schinzel H, Thürmel K (2014). Haemostasis in chronic kidney disease. Nephrology, Dialysis, Transplantation.

[35] Bhatt DL, Steg PG, Ohman EM, Hirsch AT, Ikeda Y, Mas JL (2006). International prevalence, recognition, and treatment of cardiovascular risk factors in outpatients with atherothrombosis. JAMA.

[36] Roe MT, Goodman SG, Ohman EM, Stevens SR, Hochman JS, Gottlieb S (2013). Elderly patients with acute coronary syndromes managed without revascularization: insights into the safety of long-term dual antiplatelet therapy with reduced-dose prasugrel versus standard-dose clopidogrel. Circulation.

[37] Yasuda S, Kaikita K, Akao M, Ako J, Matoba T, Nakamura M (2019). Antithrombotic Therapy for Atrial Fibrillation with Stable Coronary Disease. The New England Journal of Medicine.

[38] Lopes RD, Heizer G, Aronson R, Vora AN, Massaro T, Mehran R (2019). Antithrombotic Therapy after Acute Coronary Syndrome or PCI in Atrial Fibrillation. The New England Journal of Medicine.

[39] Gragnano F, Giacoppo D, Choi KH, Kimura T, Watanabe H, Kim HS (2026). Clopidogrel Versus Aspirin Monotherapy in Coronary Artery Disease: A Sex-Stratified Individual Patient Data Meta-Analysis of Randomized Trials. Circulation.

[40] Claassens DMF, Vos GJA, Bergmeijer TO, Hermanides RS, van 't Hof AWJ, van der Harst P (2019). A Genotype-Guided Strategy for Oral P2Y_12_ Inhibitors in Primary PCI. The New England Journal of Medicine.

[41] Pereira NL, Farkouh ME, So D, Lennon R, Geller N, Mathew V (2020). Effect of Genotype-Guided Oral P2Y12 Inhibitor Selection vs Conventional Clopidogrel Therapy on Ischemic Outcomes After Percutaneous Coronary Intervention: The TAILOR-PCI Randomized Clinical Trial. JAMA.

[42] Collet JP, Cuisset T, Rangé G, Cayla G, Elhadad S, Pouillot C (2012). Bedside monitoring to adjust antiplatelet therapy for coronary stenting. The New England Journal of Medicine.

[43] Costa F, van Klaveren D, James S, Heg D, Räber L, Feres F (2017). Derivation and validation of the predicting bleeding complications in patients undergoing stent implantation and subsequent dual antiplatelet therapy (PRECISE-DAPT) score: a pooled analysis of individual-patient datasets from clinical trials. Lancet (London, England).

[44] Urban P, Mehran R, Colleran R, Angiolillo DJ, Byrne RA, Capodanno D (2019). Defining high bleeding risk in patients undergoing percutaneous coronary intervention: a consensus document from the Academic Research Consortium for High Bleeding Risk. European Heart Journal.

[45] Gragnano F, van Klaveren D, Heg D, Räber L, Krucoff MW, Raposeiras-Roubín S (2025). Derivation and Validation of the PRECISE-HBR Score to Predict Bleeding After Percutaneous Coronary Intervention. Circulation.

[46] Fredenburgh JC, Weitz JI (2021). Factor XI as a Target for New Anticoagulants. Hamostaseologie.

[47] Sharma M, Dong Q, Hirano T, Kasner SE, Saver JL, Masjuan J (2026). Asundexian for Secondary Stroke Prevention. The New England journal of medicine.

[48] Piccini JP, Patel MR, Steffel J, Ferdinand K, Van Gelder IC, Russo AM (2025). Asundexian versus Apixaban in Patients with Atrial Fibrillation. The New England journal of medicine.

[49] Bristol-Myers Squibb (2025). Update on Phase 3 Librexia-ACS trial (sponsor press release). https://news.bms.com/news/details/2025/Update-on-Phase-3-Librexia-ACS-Trial/.

[50] Wilson SJ, Ismat FA, Wang Z, Cerra M, Narayan H, Raftis J (2018). PAR4 (Protease-Activated Receptor 4) Antagonism With BMS-986120 Inhibits Human Ex Vivo Thrombus Formation. Arteriosclerosis, Thrombosis, and Vascular Biology.

[51] Storey RF, Gurbel PA, Ten Berg J, Bernaud C, Dangas GD, Frenoux JM (2020). Pharmacodynamics, pharmacokinetics, and safety of single-dose subcutaneous administration of selatogrel, a novel P2Y12 receptor antagonist, in patients with chronic coronary syndromes. European Heart Journal.

[52] Ali ZA, Maehara A, Généreux P, Shlofmitz RA, Fabbiocchi F, Nazif TM (2016). Optical coherence tomography compared with intravascular ultrasound and with angiography to guide coronary stent implantation (ILUMIEN III: OPTIMIZE PCI): a randomised controlled trial. Lancet (London, England).

[53] Mayer K, Hein-Rothweiler R, Schüpke S, Janisch M, Bernlochner I, Ndrepepa G (2021). Efficacy and Safety of Revacept, a Novel Lesion-Directed Competitive Antagonist to Platelet Glycoprotein VI, in Patients Undergoing Elective Percutaneous Coronary Intervention for Stable Ischemic Heart Disease: The Randomized, Double-blind, Placebo-Controlled ISAR-PLASTER Phase 2 Trial. JAMA Cardiology.

[54] Pottecher J, Raffi F, Jandrot-Perrus M, Binay S, Comenducci A, Desort-Henin V (2024). Targeting GPVI with glenzocimab in COVID-19 patients: Results from a randomized clinical trial. PloS One.

[55] Lee SY, Jeong YH, Yun KH, Cho JY, Gorog DA, Angiolillo DJ (2023). P2Y_12_ Inhibitor Monotherapy Combined With Colchicine Following PCI in ACS Patients: The MACT Pilot Study. JACC. Cardiovascular Interventions.

[56] Mourikis P, Benkhoff M, Wildeis L, Barcik M, Helten C, Coman C (2025). Icosapent ethyl reduces arterial thrombosis by inhibition of cyclooxygenase-1-induced platelet reactivity. Science Translational Medicine.

